# Heparin-Induced Thrombocytopenia in Patients Undergoing Venoarterial Extracorporeal Membrane Oxygenation

**DOI:** 10.3390/jcm12010362

**Published:** 2023-01-02

**Authors:** Enzo Lüsebrink, Clemens Scherer, Leonhard Binzenhöfer, Sabine Hoffmann, Julia Höpler, Antonia Kellnar, Manuela Thienel, Dominik Joskowiak, Sven Peterß, Tobias Petzold, Simon Deseive, Ralph Hein, Stefan Brunner, Stefan Kääb, Daniel Braun, Hans Theiss, Jörg Hausleiter, Christian Hagl, Steffen Massberg, Martin Orban

**Affiliations:** 1 Medizinische Klinik und Poliklinik I, Klinikum der Universität München and DZHK (German Center for Cardiovascular Research), Partner Site Munich Heart Alliance, 81377 Munich, Germany; 2 Institut für Medizinische Informationsverarbeitung Biometrie und Epidemiologie, Klinikum der Universität München, 81377 Munich, Germany; 3 Herzchirurgische Klinik und Poliklinik, Klinikum der Universität München and DZHK (German Center for Cardiovascular Research), Partner Site Munich Heart Alliance, 81377 Munich, Germany

**Keywords:** VA-ECMO, thrombocytopenia, heparin-induced thrombocytopenia

## Abstract

**Background:** Heparin-induced thrombocytopenia (HIT) is a serious, immune-mediated adverse drug reaction to unfractionated heparin (UFH) affecting also patients undergoing venoarterial extracorporeal membrane oxygenation (VA-ECMO). Although the association between VA-ECMO support and the development of thrombocytopenia has long been known and discussed, HIT as one underlying cause is still insufficiently understood. Therefore, the purpose of this study was to further investigate the epidemiology, mortality, diagnosis, and clinical management of HIT occurring in VA-ECMO patients treated with UFH. **Methods**: We conducted a retrospective single-center study including adult patients (≥18 years) with VA-ECMO support in the cardiac intensive care unit (ICU) of the University Hospital of Munich (LMU) between January 2013 and May 2022, excluding patients with a known history of HIT upon admission. Differences in baseline characteristics and clinical outcome between excluded HIT (positive anti-platelet factor 4 (PF4)/heparin antibody test but negative functional assay) and confirmed HIT (positive anti-PF4/heparin antibody test and positive functional assay) VA-ECMO patients as well as diagnosis and clinical management of HIT were analysed. **Results**: Among the 373 patients included, anti-PF4/heparin antibodies were detected in 53/373 (14.2%) patients. Functional HIT testing confirmed HIT in 13 cases (3.5%) and excluded HIT in 40 cases (10.7%), corresponding to a prevalence of confirmed HIT of 13/373 (3.5%) [1.6, 5.3] and a positive predictive value (PPV) of 24.5% for the antibody screening test. The platelet course including platelet recovery following argatroban initiation was similar between all groups. One-month mortality in patients with excluded HIT was 14/40 (35%) and 3-month mortality 17/40 (43%), compared to 5/13 (38%) (*p* > 0.999), and 6/13 (46%) (*p* > 0.999) in patients with confirmed HIT. Neurological outcome in both groups measured by the cerebral performance category of survivors on hospital discharge was similar, as well as adverse events during VA-ECMO therapy. **Conclusions:** With a prevalence of 3.5%, HIT is a non-frequent complication in patients on VA-ECMO and was not associated with a higher mortality rate. HIT was ultimately excluded by functional essay in 75% of VA-ECMO patients with clinical suspicion of HIT and positive anti-PF4/heparin antibody test. Argatroban seems to be an appropriate and safe therapeutic option for confirmed HIT-positive patients on VA-ECMO support.

## 1. Introduction 

Venoarterial extracorporeal membrane oxygenation (VA-ECMO) has become one of the preferred devices for short-term hemodynamic support in severe cardiogenic shock stages D and E (Society for Cardiovascular Angiography and Interventions (SCAI)) [1,2]. The fundamental principle underlying temporary mechanical cardiopulmonary support is the concept of VA-ECMO as a bridge to myocardial recovery, durable mechanical circulatory support, transplantation, or refined decision-making based on the assessment of reversibility of end-organ damage and the patient’s overall prognosis [3,4]. Extracorporeal circuits, consisting of membrane oxygenators, centrifugal pumps, and cannulae, necessarily require the use of anticoagulation, usually unfractionated heparin (UFH), to minimize the risk of clotting within these components [5,6]. This in turn creates a risk for relevant bleeding complications [7,8].

Due to the steadily growing use of VA-ECMO support, it is important to pay more attention to the risks associated with this type of therapy. Among those, thrombocytopenia is of particular interest [5,9]. Previous studies showed that a platelet-reducing effect of ECMO therapy is caused by the induction of platelet aggregation on the non-endothelialised surface of the extracorporeal circuit as well as an accompanying widespread activation of the innate immune system resulting in a global generation of a proinflammatory pattern [10,11,12]. However, in addition to circuit-related thrombocytopenia, heparin-induced thrombocytopenia (HIT) is a serious differential diagnosis when platelets drop in VA-ECMO patients [13]. HIT is characterized by a decrease in platelet count of more than 50% from the highest value after the start of heparin, an onset five to ten days after the start of heparin, the presence of heparin-dependent, platelet-activating IgG antibodies, and hypercoagulability [14]. To date, HIT in VA-ECMO patients remains insufficiently understood and most studies investigating HIT occurrence are rather small studies with the inherent limitation of statistical modelling. Thus, literature findings are controversial, especially regarding the frequency and mortality of HIT in this patient cohort [13,15], but also diagnosis and clinical management of this serious, immunologically mediated adverse drug reaction to UFH [16]. Our study aims to contribute to answering these highly relevant research questions.

## 2. Methods

Study design and patient selection. The present retrospective, single-center study included ICU patients undergoing VA-ECMO treatment in the cardiac intensive care unit (ICU) of the University Hospital of Munich (LMU) between January 2013 and May 2022. All data, i.e., medical history, laboratory analysis, monitoring reports, and clinical notes, were taken from the central clinical database and detailed documentation of each patient with subsequent strict data anonymization. Clinically relevant events occurring during VA-ECMO support were recorded in a separate ICU database. Patients <18 years, experiencing ongoing pregnancy, and who had a known history of HIT were excluded. The clinical data were collected by senior clinicians. The validity and integrity of the clinical research dataset were controlled by one trained ICU physician and one senior ICU physician and by our statistical team. This is the primary analysis of these data which were exclusively compiled to investigate HIT in VA-ECMO patients.

The primary outcome variables were the prevalence of confirmed HIT during VA-ECMO treatment, in-hospital, 1-month, 3-month, and 12-month mortality rates as well as neurological outcome measured by the cerebral performance category (CPC score) of survivors at hospital discharge. Other outcomes included the time course of platelet count, the precision of HIT screening tests, specifically the HIT-4T-Score and the anti-PF4/heparin antibody test, and the HIT-associated adverse events, as well as safety and effectiveness of argatroban for alternative anticoagulation therapy in VA-ECMO patients.

Anticoagulation during VA-ECMO treatment. A standardized protocol for anticoagulation was used for all patients with an initial bolus of UFH (5000 IU) and continued intravenous UFH infusion. The dose of UFH was adapted four times daily according to activated partial thrombin time (aPTT), targeting an aPTT of 60–80 s, and clinical tolerance. If bleeding occurred, UFH therapy was adjusted or paused according to the clinical judgment of the responsible intensivists. The membrane oxygenator and circuitry were checked daily by experienced perfusionists with close monitoring of platelet counts, blood fibrinogen levels as well as signs of thrombus formation within the VA-ECMO system and significant intravascular hemolysis.

HIT diagnosis and clinical management. VA-ECMO patients were classified into three groups: HIT suspicious (positive anti-PF4/heparin antibody test), non-confirmed HIT (positive anti-PF4/heparin antibody test but negative functional assay), and confirmed HIT (positive anti-PF4/heparin antibody test and positive functional assay). At the time of HIT suspicion, the HIT-4T score as a scoring system designed to assess the pre-test probability of HIT based on the relative platelet count fall, the timing of the onset of the platelet count fall, the presence or absence of thrombosis, and the likelihood of another cause of thrombocytopenia [17] were calculated. Furthermore, the anti-PF4/heparin antibody test was performed. The combination of positive anti-PF4/heparin antibodies with at least one positive functional assay, i.e., serotonin release assay (SRA), heparin-induced platelet activation assay (HIPA), and/or platelet aggregation test (PAT) defined the confirmed HIT group. Otherwise, patients with positive anti-PF4/heparin antibodies but negative functional testing were assigned to the non-confirmed HIT cohort. If the responsible intensivist has made the decision to discontinue UFH therapy due to a relevant platelet count fall, argatroban was used as the primary alternative anticoagulant.

Ethics Approval. The study was conducted in accordance with the Declaration of Helsinki and German data protection laws. All data were extracted from the LMUshock registry. The latter is registered at the WHO International Clinical Trials Registry Platform (DRKS00015860) and was approved by the local ethics committee (IRB number: 18-001).

Statistical analysis. Statistical analysis was performed using R^®^ (version 4.0.3). Continuously distributed variables were reported as medians with interquartile ranges (25th and 75th percentile) and categorical variables were reported as absolute numbers and percentages. Patient characteristics were compared using Wilcoxon Rank-Sum tests for continuous variables and Fisher’s exact test or the Chi-square test for categorical variables. All tests were 2-tailed, and *p*-values < 0.05 were considered significant.

## 3. Results

**Figure 1:**
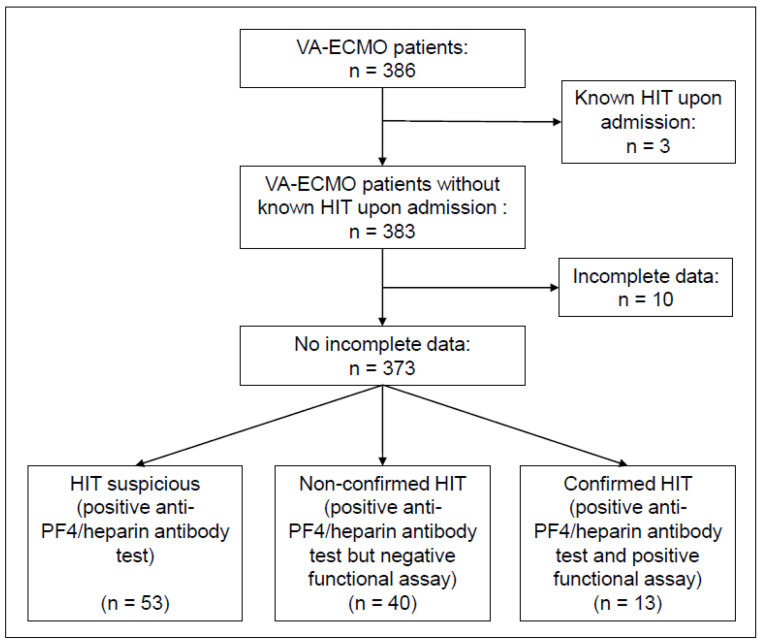
Flow diagram depicting patient selection.

Study population. During the study period, a total of 386 patients received VA-ECMO support at the cardiac ICU of the University Hospital of Munich (LMU). Of these, three were excluded due to the presence of HIT upon admission and ten due to incomplete data (Figure 1). Detailed baseline characteristics and information on ICU treatment are presented in Table 1. The median age at admission was 59.0 years with 306/373 (82%) being male. There were no significant differences in cardiovascular risk factors and morbidity at admission between the confirmed and excluded HIT groups. Median initial haemoglobin levels were lower in the confirmed HIT group (12.9 g/dL vs. 9.7 g/dL, *p* = 0.038), while the platelet count was similar (200.0 G/L vs. 176.0 G/L, *p* = 0.358). Cardiogenic shock due to acute myocardial infarction was the most frequent indication for VA-ECMO (60%), followed by cardiomyopathy (19%), myocarditis (6%), and cardiac arrhythmia (6%). In 29% of total cases, renal replacement therapy was required and in 16%, a microaxial flow pump was implanted in addition to VA-ECMO. Usage of norepinephrine was more frequent in the confirmed HIT group (73% vs. 100%, *p* = 0.047).

HIT prevalence. Following HIT suspicion, anti-PF4/heparin antibodies were detected in 53/373 patients (14.2% [10.7–17.8]), all of whom underwent subsequent functional testing. HIT was confirmed in 13/373 cases (3.5% [1.6–5.3]), while in 40/373 cases (10.7% [7.6–13.9]), HIT was excluded, corresponding to a positive predictive value (PPV) of 24.5% (13/53) for the HIT antibody screening test. 

HIT diagnosis and management. Detailed information on HIT diagnosis and management is summarized in Table 2. The median duration of unfractionated heparin (UFH) therapy before anti-PF4/heparin antibody testing was five days [3,10] and eight days [2,11] in patients with excluded and confirmed HIT, respectively (*p* = 0.959). Functional assay testing was done six days [3,10] and eight days [2,11] after starting UFH therapy, respectively (*p* = 0.959). The median HIT-4T score at the time of HIT suspicion was 4 [3,5] and 5 [4,6], respectively (*p* = 0.054). Argatroban was used in all patients for alternative anticoagulation. Additional information regarding HIT prevalence, diagnosis, and management in male compared to female patients are presented in Supplementary Table S1.

**Figure 2:**
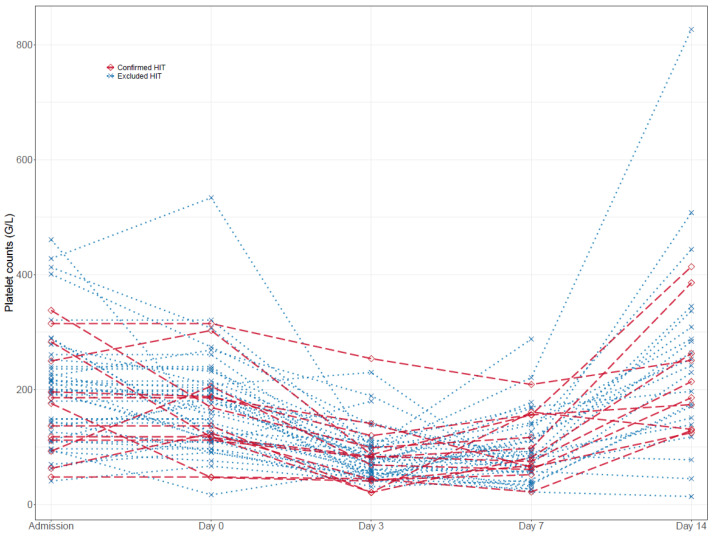
Time course of platelet counts in excluded and confirmed HIT patients.

Platelet counts during VA-ECMO therapy. Platelet counts at admission, day seven, and day fourteen were largely similar among all groups. However, the maximum platelet count under UFH therapy was significantly higher in patients with excluded HIT compared to confirmed HIT (286 G/L [192, 360] vs. 176 G/L [124, 206], *p* = 0.012), but the minimum platelet count under UFH (44 [33, 71] vs. 50 [26, 65], *p* = 0.885) was not (Table 2). Individual platelet count courses are presented in Table 3 and Figure 2. Both patient groups with excluded and confirmed HIT experienced a significant platelet drop between admission and day seven of VA-ECMO therapy (−103.5 G/L [−164.2, −50.0], *p* < 0.001; −40.0 G/L [−108.0, −26.0], *p* = 0.040), while thrombocyte count recovered at day fourteen under continued UFH or argatroban therapy, respectively (55.0 G/L [−50.0, 148.0], *p* = 0.246; 37.5 G/L [−63.2, 132.0], *p* = 0.264). Notably, there were no significant differences between relative platelet count changes in patients with excluded and confirmed HIT (admission/day 0, *p* = 0.444; admission/day 3, *p* = 0.326; admission/day 7, *p* = 0.125; admission/day 14, *p* = 0.983).

Outcome. The median length of ICU stays [d] (10.5 vs. 20.6, *p* = 0.018) and duration of mechanical ventilation [h] (189 vs. 398, *p* = 0.015) was significantly lower in excluded HIT compared to confirmed HIT. However, in-hospital mortality (43% vs. 38%, *p* > 0.999) and mortality after one month (35% vs. 38%, *p* > 0.999), three months (43% vs. 46%, *p* > 0.999), and twelve months (53% vs. 46%, *p* = 0.938), as well as a neurological outcome at hospital discharge was similar in patients with excluded and confirmed HIT (Table 4). Gender-specific differences in outcomes are presented in Supplementary Table S2.

Adverse events during VA-ECMO therapy. Bleeding was the most frequent complication observed during VA-ECMO therapy without significant differences between excluded and confirmed HIT group (BARC-3 bleeding: 28% vs. 31%, *p* > 0.999), followed by hemolysis (20% vs. 23%, *p* > 0.999), arterial thrombosis (10% vs. 15%, *p* = 0.627), and venous thrombosis (8% vs 15%, *p* = 0.586). Device malfunctions requiring system exchanges were less common in both the excluded and confirmed HIT group (Table 5). Gender-specific differences in adverse events during VA-ECMO therapy are presented in Supplementary Table S3.

## 4. Discussion

In this comprehensive single-center analysis, HIT was confirmed in 3.5% of patients treated with VA-ECMO, which corresponds to previously reported prevalence rates ranging from 0.36% to 8.3% and the estimated overall prevalence of up to 5% in adults receiving UFH [13,15,16,18,19,20,21,22,23,24]. A recent meta-analysis has found thrombocytopenia to occur in 23.2% of VA-ECMO-treated patients [19]. Identifying patients with HIT and other treatable causes is a major challenge, considering the wide array of differential diagnoses underlying thrombocytopenia in patients receiving extracorporeal circulatory support and concomitant UFH therapy. Standard screening tools such as the original HIT-4T Score, the Lilo-Le Louet Score, and anti-PF4/heparin antibody tests have failed to yield high PPVs in this subset of ICU patients when HIT is suspected [20,22]. Modifications of the HIT-4T Score have shown better accuracy in a general ICU population and during VA-ECMO support regarding the rule-out of HIT [25,26]. In the presented cohort, the PPV of the anti-PF4/heparin antibody test was 24.5%, as compared to 53% reported by Kimmoun et al., 33.3% by Sullivan et al., and 8.5% by Vayne et al. [13,20,27]. The HIT 4-T Score was numerically higher for patients with confirmed HIT, but this did not reach significance, considering that it has not been designed or assessed in the specific context of mechanical circulatory support. Individual platelet count courses did not differentiate patients with confirmed and excluded HIT either, which too was described previously by Kimmoun et al. as well as Zaaqoq et al. [13,22]. On the other hand, we have found significantly lower maximum platelet counts as well as lower initial hemoglobin levels in HIT-confirmed cases. These factors could potentially contribute to the development of more accurate prediction models to better identify patients with an a priori increased risk of HIT for whom close monitoring starting directly from the beginning of VA-ECMO treatment with optimal awareness and readiness for complication management is required. Considering the gravity of potential complications if HIT is underdiagnosed, clinicians should be advised to immediately initiate functional testing in all patients with the positive antibody screening test and high clinical suspicion of HIT. Early identification of HIT allows for better decision-making regarding adjustment of antithrombotic treatment, anticoagulation, and transfusion regimes and helps to prevent complications.

Furthermore, thrombocytopenia during VA-ECMO may be aggravated by additionally acquired platelet dysfunction owing to constant shear stress by the ECMO pump and coagulation disorders resulting from contact with the large extracorporeal circuit [28]. The molecular mechanism of this phenomenon is incompletely understood but may be related to reduced glycoprotein (GP) Ibα (receptor for VWF) and GPVI (receptor for collagen) [29]. Extended platelet function and coagulation testing could allow for better identification of patients with coagulation disorders, including platelet dysfunctions, and should be considered for future studies [30].

Mortality rates within the first year after cardiogenic shock were 46% in patients with confirmed HIT and 53% when HIT was excluded. In fact, the comparison of the two groups did not show significant differences for all evaluated time points. This finding is interesting since ICU stay and mechanical ventilation times nearly doubled in confirmed HIT cases. In our analysis, this was neither due to hemorrhagic or thromboembolic complications, nor transfusion requirements, nor insufficient platelet regeneration following the switch to an anticoagulant regimen. In a large retrospective analysis including twenty French VA-ECMO centers, mortality rates at 90 days of 50.0% and 33.3% have been found for excluded and confirmed HIT patients, respectively, without a significant difference between groups (*p* = 0.48) [13]. Median ICU stay was numerically longer in the confirmed HIT group (26.5 days vs. 41.0 days, *p* = 0.22) and overall, it was longer compared to the cohort presented here [13]. A meta-analysis evaluating HIT during VA-ECMO therapy by Choi et al. found a 46.7% mortality rate in patients with confirmed HIT at the end of follow-up [31]. Of note, the protocol for HIT confirmation in included publications was not consistent. Indeed, the authors found a much higher rate (53%) of individuals who suffered thromboembolic adverse events during VA-ECMO, necessitating device or circuit change in two patients [31]. Recently, Zaaqoq and colleagues published a single-center study including 47% post-cardiotomy shock patients that showed comparable mortality in patients with positive vs. negative functional HIT testing [22]. In two other retrospective trials including patients who underwent veno-venous and veno-arterial ECMO support, mortality rates were similar, as well [23,32]. Conclusively, the diagnosis of HIT following VA-ECMO therapy does not seem to influence overall survival, despite being associated with a longer length of ICU stay. The data presented here do not provide evidence for a relevant increase in HIT-specific adverse events under adequate anticoagulation therapy using argatroban.

Here, the use of argatroban has been shown to be an effective alternative anticoagulant in patients with suspected and confirmed HIT [13,27,33,34]. Of note, Althaus et al. reported two cases of thromboembolic events after adapting anticoagulation to argatroban in patients with confirmed HIT [21]. The direct thrombin-inhibitor bivalirudin has also been used but so far there is no study focusing specifically on HIT as an indication for bivalirudin in adult VA-ECMO patients. [31,35,36,37]. In our center, where argatroban was the preferred anticoagulant agent, we observed sufficient platelet recovery after adaptation of the anticoagulant management as did Rougé et al. [34].

**Limitations:** The limitations inherent to this observational study mainly result from a lack of randomization and blinding. Although our investigation on HIT in VA-ECMO patients offers one of the most comprehensive datasets published to date, generalizability may be affected owing to differences in VA-ECMO patient management as well as HIT diagnostic process and management towards other centers. In addition, some patients included in the analysis may have suffered from coagulation disorders at baseline or even from unreported previously confirmed HIT, which could have influenced the presented results. However, these disorders usually have relevant clinical visibility once they are established, and routine screening and functional testing for all of these underlying conditions would not be practicable anyway. Finally, the diagnostic algorithm used at our center implies a verification bias for both the HIT-4T score and the anti-PF4/heparin screening test, which makes a fuller understanding of predictive values and overall diagnostic accuracy infeasible.

## 5. Conclusions 

The present study, based on the analysis of 373 ICU patients, showed that HIT with a prevalence of 3.5% is a non-frequent complication in adult patients treated with VA-ECMO and was not associated with a significantly higher mortality rate. The positive predictive value of the anti-PF4/heparin antibody screening test was 25%. In patients with confirmed HIT, argatroban seemed to be an appropriate and safe anticoagulant option during VA-ECMO support. Future studies should aim at developing more precise prediction models (e.g., using haemoglobin levels) to prevent HIT-associated complications as well as the overuse of costly diagnostic tests and non-standard anticoagulation protocols.

**Take-home message:**

- Prevalence of HIT is 3.5% in patients on VA-ECMO treatment.
- HIT was not associated with a significantly higher mortality rate.
- Argatroban seems to be an appropriate and safe therapeutic option for confirmed HIT-positive patients undergoing VA-ECMO support.

## Figures and Tables

**Table 1:** Baseline characteristics and ICU treatment.

Characteristics		Overall	Patients with HIT Suspicion	Patients with Excluded HIT	Patients with Confirmed HIT	*p*-Value (III vs. IV)
		(n = 373) (I)	(n = 53) (II)	(n = 40) (III)	(n = 13) (IV)	
**Demographics**						
Age [years], median [IQR]		59.0 [51.0, 67.0]	59.0 [44.0, 63.0]	59.0 [45.5, 64.2]	56.0 [35.0, 63.0]	0.45
Sex [male], n (%)		306 (82)	44 (83)	32 (80)	12 (92)	0.424
Body mass index [kg/m²], median [IQR]		26.8 [24.5, 29.4]	26.8 [24.2, 29.6]	26.5 [23.8, 29.7]	27.8 [25.9, 29.4]	0.357
**Morbidity at admission**						
History of stroke, n (%)		36 (10)	4 (8)	4 (10)	0 (0)	0.561
History of cancer, n (%)		17 (5)	2 (4)	2 (5)	0 (0)	>0.999
History of atrial fibrillation, n (%)		50 (13)	6 (11)	5 (13)	1 (8)	>0.999
Coronary artery disease, n (%)		111 (30)	13 (25)	11 (28)	2 (15)	0.48
Previous myocardial infarction, n (%)		71 (19)	7 (13)	6 (15)	1 (8)	0.667
Previous percutaneous coronary intervention, n (%)		71 (19)	7 (13)	6 (15)	1 (8)	0.424
Previous coronary artery bypass graft, n (%)		16 (4)	2 (4)	2 (5)	0 (0)	>0.999
Peripheral artery disease, n (%)		27 (7)	3 (6)	3 (8)	0 (0)	0.567
Hypertension, n (%)		199 (53)	28 (53)	24 (60)	4 (31)	0.109
Diabetes mellitus, n (%)		82 (22)	10 (19)	9 (23)	1 (8)	0.419
Chronic renal disease, n (%)		28 (8)	7 (13)	7 (18)	0 (0)	0.174
Pre-cannulation SAPS II score, median [IQR]		75.0 [67.0, 81.0]	71.0 [63.0, 77.0]	71.5 [63.8, 78.0]	66.0 [62.0, 76.0]	0.469
Pre-cannulation SOFA score, median [IQR]		13.0 [11.0, 15.0]	13.0 [11.0, 15.0]	13.0 [10.0, 15.0]	13.0 [11.0, 15.0]	0.37
Cardiac arrest, n (%)		258 (69)	29 (55)	19 (48)	10 (77)	0.108
Extracorporeal cardiopulmonary resuscitation, n (%)		65 (17)	6 (11)	5 (13)	1 (8)	>0.999
**Medication at admission**						
Aspirin, n (%)		99 (27)	14 (26)	13 (33)	1 (8)	0.145
P2Y12-inhibitor, n (%)		86 (23)	9 (17)	8 (20)	1 (8)	0.424
Aspirin + P2Y12-inhibitor, n (%)		56 (15)	7 (13)	6 (15)	1 (8)	0.667
**Gas exchange at ICU admission**						
pH, median [IQR] *		7.3 [7.3, 7.4]	7.4 [7.3, 7.5]	7.4 [7.3, 7.5]	7.4 [7.3, 7.4]	0.82
Arterial PaO_2_ [mmHg], median [IQR] *		132.0 [90.0, 253.0]	119.0 [93.3, 192.0]	112.0 [92.6, 198.2]	153.0 [109.0, 175.0]	0.788
Arterial PaCO_2_ [mmHg], median [IQR] *		33.9 [29.1, 40.0]	32.1 [27.9, 39.3]	31.1 [27.7, 37.8]	34.8 [29.7, 40.3]	0.584
**Laboratory values at ICU admission**						
Lactate [mmol/L], median [IQR] *		6.8 [2.7, 11.1]	3.3 [1.7, 7.9]	3.3 [1.7, 8.0]	3.9 [1.5, 6.3]	>0.999
Creatinine [mg/dl], median [IQR] *		1.5 [1.2, 2]	1.5 [1.2, 2.2]	1.5 [1.2, 2.2]	1.6 [1.2, 2.2]	0.959
Bilirubin [mg/dl], median [IQR] *		0.9 [0.5, 1.6]	1.1 [0.8, 2.4]	1.3 [0.7, 2.5]	0.9 [0.8, 1.4]	0.326
Aspartate aminotransferase [U/I], median [IQR] *		338.0 [125.0, 936.0]	322.0 [118.0, 672.0]	325.5 [124.0, 645.8]	288.0 [65.0, 1002.0]	0.894
Alanine aminotransferase [U/I], median [IQR] *		147.0 [67.0, 398.0]	121.0 [73.0, 508.0]	122.5 [75.2, 491.2]	93.0 [53.0, 508.0]	0.877
Hemoglobin [g/dl], median [IQR] *		11.7 [9.6, 13.6]	12.2 [9.8, 14.4]	12.9 [10.4, 14.6]	9.7 [9.2, 13.1]	0.038
Platelet count [G/L], median [IQR] *		189.0 [127.0, 240.0]	197.0 [118.0, 250.0]	200.0 [132.5, 243.0]	176.0 [112.0, 250.0]	0.358
aPTT [sec], median [IQR] *		63.0 [33.0, 160.0]	46.0 [33.0, 115.0]	46.0 [32.5, 108.5]	40.0 [35.0, 160.0]	0.561
INR, median [IQR] *		1.8 [1.3, 2.6]	1.7 [1.4, 2.3]	1.8 [1.4, 2.2]	1.5 [1.3, 3.4]	0.686
**VA-ECMO set-up**						
VA-ECMO indication	ST-elevation myocardial infarction, n (%)	157 (42)	19 (36)	15 (38)	4 (31)	0.749
	Non-ST segment elevation myocardial infarction, n (%)	69 (18)	4 (8)	2 (5)	2 (15)	0.249
	Cardiomyopathy, n (%)	71 (19)	17 (32)	13 (33)	4 (31)	>0.999
	Myocarditis, n (%)	22 (6)	6 (11)	4 (10)	2 (15)	0.627
	Cardiac arrhythmia, n (%)	22 (6)	4 (8)	4 (10)	0 (0)	0.561
	Pulmonary embolism, n (%)	7 (2)	1 (2)	0 (0)	1 (8)	0.245
	Septic shock, n (%)	3 (0.8)	1 (2)	1 (3)	0 (0)	>0.999
	Others, n (%)	22 (6)	1 (2)	1 (3)	0 (0)	>0.999
Peripheral cannulation, n (%)		373 (100)	53 (100)	40 (100)	13 (100)	1
Intra-aortic balloon pump therapy, n (%)		0 (0)	0 (0)	0 (0)	0 (0)	1
Impella therapy, n (%)		58 (16)	14 (26)	11 (28)	3 (23)	>0.999
Mechanical ventilation, n (%)		335 (90)	45 (85)	33 (83)	12 (92)	0.662
Renal replacement therapy, n (%)		108 (29)	13 (25)	9 (23)	4 (31)	0.712
Vasopressors	Epinephrine, n (%)	209 (56)	19 (36)	16 (40)	3 (23)	0.334
	Norepinephrine, n (%)	285 (76)	42 (79)	29 (73)	13 (100)	0.047
	Dobutamine, n (%)	56 (15)	20 (38)	16 (40)	4 (31)	0.744
	Vasopressin, n (%)	47 (13)	6 (11)	4 (10)	2 (15)	0.627
Red blood cell transfusion [units], median [IQR]		6 [2, 10]	6 [4, 11]	6 [2, 10]	8 [5, 13]	0.228
Platelet transfusions [units], median [IQR]		0 [0, 0]	0 [0, 0]	0 [0, 0]	0 [0, 0]	0.937
Plasma transfusions [units], median [IQR]		6 [2, 12]	8 [4, 13]	8 [4, 12.2]	11 [4, 17]	0.52
Total duration of mechanical ventilation [h], median [IQR]		132 [23, 313]	220 [84, 398]	189 [53, 364]	398 [304, 617]	0.015
Total duration of VA-ECMO treatment [h], median [IQR]		89 [39, 144]	157 [90, 176]	149 [88, 169]	176 [99, 215]	0.13

Baseline characteristics and ICU treatment. aPTT, activated partial thromboplastin time; ECMO, extracorporeal membrane oxygenation; h, hours; HIT, heparin-induced thrombocytopenia; ICU, intensive care unit; INR, international normalized ratio; IQR, interquartile range; n, number of patients; PaCO2, partial pressure of carbon dioxide; PaO2, partial pressure of oxygen; SAPS II score, simplified acute physiology II score; SOFA score, sequential organ failure assessment score; VA-ECMO, venoarterial extracorporeal membrane oxygenation; * First value measured on ICU.

**Table 2:** HIT diagnosis and management.

Characteristics		Patients with HIT Suspicion	Patients with Excluded HIT	Patients with Confirmed HIT	*p*-Value
		(n = 53) (II)	(n = 40) (III)	(n = 13) (IV)	(III vs. IV)
**HIT Diagnosis**					
Continuous unfractionated heparin therapy before HIT suspicion, n (%)		53 (100)	40 (100)	13 (100)	1
Heparin-bonded VA-ECMO circuit, n (%)		53 (100)	40 (100)	13 (100)	1
Duration of heparin therapy before anti-PF4/heparin antibody testing [d], median [IQR]		5 [3, 10]	5 [3, 10]	8 [2, 11]	0.959
Positive anti-PF4/heparin antibody testing, n (%)		53 (100)	40 (100)	13 (100)	
Duration of heparin therapy before		7 [3, 10]	6 [3, 10]	8 [2, 11]	0.959
Confirmed HIT-functional assay [d], median [IQR]					
Positive HIT-functional assay, n (%)		13 (25)	0 (0)	13 (100)	
HIT-4T-Score, median [IQR]		4 [4, 5]	4 [3, 5]	5 [4, 6]	0.054
**HIT Management**					
Anticoagulant therapy after HIT confirmation	Argatroban, n (%)			13 (100)	
	Danaparoid, n (%)			0 (0)	
	Bivalirudin, n (%)			0 (0)	
Duration of heparin therapy before anticoagulation change [d], median [IQR]				8 [2, 11]	
**Platelet Counts**					
Platelet count at admission [G/L], median [IQR]		197 [118, 250]	200 [133, 243]	176 [112, 250]	0.358
Platelet count at the beginning of VA-ECMO therapy [G/L], median [IQR]		164 [114, 208]	170 [113, 210]	137 [117, 188]	0.605
Platelet count at day 3 of VA-ECMO therapy [G/L], median [IQR]		75 [50, 100]	73 [53, 101]	82 [43, 99]	0.8
Platelet count at day 7 of VA-ECMO therapy [G/L], median [IQR]		81 [58, 123]	81 [56, 117.2]	82 [65, 156]	0.513
Platelet count at day 14 of VA-ECMO therapy [G/L], median [IQR]		230 [145, 299]	242 [151, 309]	200 [142, 260]	0.693
Minimum platelet count during heparin therapy [G/L], median [IQR]		45 [33, 71]	44 [33, 71]	50 [26, 65]	0.885
Maximum platelet count during heparin therapy [G/L], median [IQR]		252 [176, 303]	286 [192, 360]	176 [124, 206]	0.012

HIT diagnosis and management. D, days; HIT, heparin-induced thrombocytopenia; IQR, interquartile range; n, number of patients; PF4, platelet factor 4; VA-ECMO, venoarterial extracorporeal membrane oxygenation.

**Table 3:** Time course of platelet counts.

Date 1	Date 2	Patients with Excluded HIT (n = 40)		Patients with Confirmed HIT (n = 13)	
		Median [IQR] of	*p*-Value for Pairwise	Median [IQR] of	*p*-Value for Pairwise
		Change in Platelet Count	Comparison	Change in Platelet Count	Comparison
			Date 1 vs. Date 2		Date 1 vs. Date 2
Admission	Beginning of VA-ECMO therapy	0.0 [−65.8, 0.0]	0.13	0.0 [−8.0, 0.0]	0.681
Admission	Day 3 of VA-ECMO	−120.0 [−174.5, −51.0]	<0.001	−76.0 [−135.0, −30.0]	0.008
Admission	Day 7 of VA-ECMO	−103.5 [−164.2, −50.0]	<0.001	−40.0 [−108.0, −26.0]	0.04
Admission	Day 14 of VA-ECMO	55.0 [−50.0, 148.0]	0.246	37.5 [−63.2, 132.0]	0.264

Time course of platelet counts. HIT, heparin-induced thrombocytopenia; IQR, interquartile range; VA-ECMO, venoarterial extracorporeal membrane oxygenation.

**Table 4:** Outcome of VA-ECMO treatment.

Characteristics		Overall	Patients with HIT Suspicion	Patients with Excluded HIT	Patients with Confirmed HIT	*p*-Value (III vs. IV)
		(n = 373) (I)	(n = 53) (II)	(n = 40) (III)	(n = 13) (IV)	
Total ICU length of stay [d], median [IQR]		8.9 [3.0, 16.0]	14.6 [8.2, 22.3]	10.5 [7.1, 19.4]	20.6 [16.7, 30.8]	0.018
Total hospital length of stay [d], median [IQR]		13.8 [4.7, 25.3]	26.3 [13.3, 49.3]	26.1 [9.5, 48.3]	28 [20.8, 51.8]	0.468
Hospital mortality, n (%)		213 (57)	22 (42)	17 (43)	5 (38)	>0.999
1-month mortality, n (%)		203 (54)	19 (36)	14 (35)	5 (38)	>0.999
3-month mortality, n (%)		222 (60)	23 (43)	17 (43)	6 (46)	>0.999
1-year mortality, n (%)		239 (64)	27 (51)	21 (53)	6 (46)	0.938
Cerebral performance category of survivors at hospital discharge	CPC1, n (%)	19 (12)	5 (16)	4 (17)	1 (13)	>0.999
	CPC2, n (%)	37 (23)	4 (13)	3 (13)	1 (13)	>0.999
	CPC3, n (%)	75 (47)	13 (25)	10 (43)	3 (38)	>0.999
	CPC4, n (%)	29 (18)	9 (29)	6 (26)	3 (38)	0.672

Outcome of VA-ECMO treatment. CPC, cerebral performance category; HIT, heparin-induced thrombocytopenia; ICU, intensive care unit; n, number of patients; VA-ECMO, venoarterial extracorporeal membrane oxygenation.

**Table 5:** Adverse events during VA-ECMO therapy.

Characteristics		Overall	Patients with HIT Suspicion	Patients With Excluded HIT	Patients with Confirmed HIT	*p*-Value (III vs. IV)
		(n = 373) (I)	(n = 53) (II)	(n = 40) (III)	(n = 13) (IV)	
**Adverse Events during VA-ECMO Therapy**						
Hemorrhage	BARC 3, n (%)	126 (34)	15 (28)	11 (28)	4 (31)	>0.999
	BARC 4, n (%)	1 (0.3)	0 (0)	0 (0)	0 (0)	1
	BARC 5, n (%)	13 (3)	1 (2)	1 (3)	0 (0)	>0.999
Stroke, n (%)		16 (4)	2 (4)	1 (3)	1 (8)	0.434
Hemolysis, n (%)		48 (13)	11 (21)	8 (20)	3 (23)	>0.999
Myocardial infarction, n (%)		9 (2)	0 (0)	0 (0)	0 (0)	1
Arterial thrombosis, n (%)		21 (6)	6 (11)	4 (10)	2 (15)	0.627
Venous thrombosis, n (%)		14 (4)	5 (9)	3 (8)	2 (15)	0.586
Device-related peripheral ischemic complications, n (%)		18 (5)	0 (0)	0 (0)	0 (0)	1
Device malfunction, n (%)		5 (1)	0 (0)	0 (0)	0 (0)	1
VA-ECMO oxygenator exchange, n (%)		7 (2)	2 (4)	2 (5)	0 (0)	>0.999
VA-ECMO circuit exchange, n (%)		13 (3)	6 (11)	5 (13)	1 (8)	>0.999
VA-ECMO oxygenator and circuit exchange, n (%)		4 (1)	1 (2)	1 (3)	0 (0)	>0.999

Adverse events during VA-ECMO therapy. BARC, bleeding academy research consortium; HIT, heparin-induced thrombocytopenia; n, number of patients; VA-ECMO, venoarterial extracorporeal membrane oxygenation.

## Data Availability

The data presented in this study are available on request from the corresponding author. The data are not publicly available due to ethical restrictions and legal constraints.

## Supplementary Materials

The following supporting information can be downloaded at: https://www.mdpi.com/article/10.3390/jcm12010362/s1, Table S1: HIT diagnosis and management differentiated by gender; Table S2: Outcome of VA-ECMO treatment differentiated by gender; Table S3: Adverse events during VA-ECMO therapy differentiated by gender.
