# Supplementary material for: Heparin-Induced Thrombocytopenia in Patients Undergoing Venoarterial Extracorporeal Membrane Oxygenation
Source: J Clin Med. 2023 Jan 2;12(1):362. doi: 10.3390/jcm12010362 (PMC9821297; doi:10.3390/jcm12010362)
Supplement: Supplementary file 1 [file jcm-12-00362-s001.zip › jcm-2092741-supplementary.pdf]

Supplementary Tables

Supplementary Table S1: HIT diagnosis and management differentiated by gender. D, days; HIT, heparin-induced thrombocytopenia; IQR, interquartile range; n, number of patients; PF4, platelet factor 4; VA-ECMO, venoarterial extracorporeal membrane oxygenation.

Supplementary Table S1: HIT diagnosis and management differentiated by gender.					
**Sex: male**					
**Characteristics**		**Patients with HIT suspicion (n = 44) (II)**	**Patients with excluded HIT (n = 32) (III)**	**Patients with confirmed HIT (n = 12) (IV)**	**p-value (III vs. IV)**
**HIT diagnosis**					
Continuous unfractionated heparin therapy before HIT suspicion, n (%)		44 (100)	32 (100)	12 (100)	
Heparin-bonded VA-ECMO circuit, n (%)		44 (100)	32 (100)	12 (100)	
Duration of heparin therapy before anti-PF4/heparin antibody testing [d], median [IQR]		5.0 [2.0, 10.0]	5.0 [2.8, 8.0]	8.5 [2.0, 11.0]	0.474
Positive anti-PF4/heparin antibody testing, n (%)		44 (100)	32 (100)	12 (100)	
Duration of heparin therapy before Confirmed HIT-functional assay [d], median [IQR]		7.0 [2.0, 10.0]	6.0 [2.8, 8.5]	8.5 [2.0, 11.0]	0.499
Positive HIT-functional assay, n (%)		12 (27)	0 (0)	12 (100)	
HIT-4T-Score, median [IQR]		4.0 [4.0, 5.0]	4.0 [3.8, 5.0]	5.0 [4.0, 6.0]	0.027
**HIT Management**					
Anticoagulant therapy after HIT confirmation	Argatroban, n (%)			12 (100)	
	Danaparoid, n (%)			0 (0)	
	Bivalirudin, n (%)			0 (0)	
Duration of heparin therapy before anticoagulation change [d], median [IQR]				8.5 [2.0, 11.0]	
**Platelet counts**					
Platelet count at admission [G/l], median [IQR]		196.5 [116.5, 250.5]	198.0 [122.5, 243.0]	181.0 [116.5, 258.2]	0.742
Platelet count at the beginning of VA-ECMO therapy [G/l], median [IQR]		166.5 [115.5, 203.0]	169.5 [112.8, 203.5]	153.0 [117.8, 192.5]	>0.999
Platelet count at day 3 of VA-ECMO therapy [G/l], median [IQR]		81.0 [49.5, 102.0]	77.0 [53.5, 102.0]	83.0 [42.5, 104.2]	0.925
Platelet count at day 7 of VA-ECMO therapy [G/l], median [IQR]		81.0 [59.5, 124.5]	78.0 [57.2, 111.8]	90.0 [67.2, 156.0]	0.221
Platelet count at day 14 of VA-ECMO therapy [G/l], median [IQR]		240.5 [166.0, 293.2]	253.0 [161.0, 298.5]	214.0 [174.0, 263.0]	0.953
Minimum platelet count under heparin therapy [G/l], median [IQR]		47.0 [35.2, 68.8]	45.5 [35.2, 68.8]	53.5 [37.2, 72.0]	0.772
Maximum platelet count under heparin therapy [G/l], median [IQR]		246.0 [175.5, 300.0]	273.0 [183.8, 307.5]	181.0 [151.8, 225.2]	0.045
**Sex: female**					
**Characteristics**		**Patients with HIT suspicion (n = 9) (II)**	**Patients with excluded HIT (n = 8) (III)**	**Patients with confirmed HIT (n = 1) (IV)**	**p-value (III vs. IV)**
**HIT diagnosis**					

<table>
<tr><td colspan="2">Continuous unfractionated heparin therapy before HIT suspicion, n (%)</td><td>9 (100)</td><td>8 (100)</td><td>1 (100)</td><td>n.a.</td></tr>
<tr><td colspan="2">Heparin-bonded VA-ECMO circuit, n (%)</td><td>9 (100)</td><td>8 (100)</td><td>1 (100)</td><td>n.a.</td></tr>
<tr><td colspan="2">Duration of heparin therapy before anti-PF4/heparin antibody testing [d], median [IQR]</td><td>4.0 [4.0, 12.0]</td><td>7.0 [4.0, 13.0]</td><td>1</td><td>n.a.</td></tr>
<tr><td colspan="2">Positive anti-PF4/heparin antibody testing, n (%)</td><td>9 (100)</td><td>8 (100)</td><td>1 (100)</td><td>n.a.</td></tr>
<tr><td colspan="2">Duration of heparin therapy before Confirmed HIT-functional assay [d], median [IQR]</td><td>4.0 [4.0, 12.0]</td><td>7.0 [4.0, 13.0]</td><td>1</td><td>n.a.</td></tr>
<tr><td colspan="2">Positive HIT-functional assay, n (%)</td><td>1 (11)</td><td>0 (0)</td><td>1 (100)</td><td>n.a.</td></tr>
<tr><td colspan="2">HIT-4T-Score, median [IQR]</td><td>4.0 [3.0, 4.0]</td><td>4.0 [3.0, 4.2]</td><td>3</td><td>n.a.</td></tr>
<tr><td colspan="6">HIT Management</td></tr>
<tr><td rowspan="3">Anticoagulant therapy after HIT confirmation</td><td>Argatroban, n (%)</td><td></td><td></td><td>1 (100)</td><td></td></tr>
<tr><td>Danaparoid, n (%)</td><td></td><td></td><td>0 (0)</td><td></td></tr>
<tr><td>Bivalirudin, n (%)</td><td></td><td></td><td>0 (0)</td><td></td></tr>
<tr><td colspan="2">Duration of heparin therapy before anticoagulation change [d], median [IQR]</td><td></td><td></td><td>1</td><td></td></tr>
<tr><td colspan="6">Platelet counts</td></tr>
<tr><td colspan="2">Platelet count at admission [G/l], median [IQR]</td><td>214.0 [142.0, 236.0]</td><td>214.5 [181.0, 280.2]</td><td>48</td><td>n.a.</td></tr>
<tr><td colspan="2">Platelet count at the beginning of VA-ECMO therapy [G/l], median [IQR]</td><td>142.0 [111.0, 236.0]</td><td>178.5 [120.0, 253.8]</td><td>48</td><td>n.a.</td></tr>
<tr><td colspan="2">Platelet count at day 3 of VA-ECMO therapy [G/l], median [IQR]</td><td>62.0 [51.0, 83.0]</td><td>67.5 [54.0, 85.2]</td><td>45</td><td>n.a.</td></tr>
<tr><td colspan="2">Platelet count at day 7 of VA-ECMO therapy [G/l], median [IQR]</td><td>90.0 [26.0, 119.0]</td><td>91.5 [35.8, 131.8]</td><td>22</td><td>n.a.</td></tr>
<tr><td colspan="2">Platelet count at day 14 of VA-ECMO therapy [G/l], median [IQR]</td><td>197.0 [134.0, 375.0]</td><td>219.5 [153.5, 441.5]</td><td>129</td><td>n.a.</td></tr>
<tr><td colspan="2">Minimum platelet count under heparin therapy [G/l], median [IQR]</td><td>32.0 [24.0, 73.0]</td><td>34.5 [28.2, 76.0]</td><td>24</td><td>n.a.</td></tr>
<tr><td colspan="2">Maximum platelet count under heparin therapy [G/l], median [IQR]</td><td>288.0 [194.0, 413.0]</td><td>317.0 [225.5, 521.5]</td><td>48</td><td></td></tr>
</table>

Supplementary Table S2. Outcome of VA-ECMO treatment differentiated by gender. CPC, cerebral performance category; HIT, heparin-induced thrombocytopenia; ICU, intensive care unit; n, number of patients; VA-ECMO, venoarterial extracorporeal membrane oxygenation.

**Supplementary Table S2: Outcome of VA-ECMO treatment differentiated by gender**					
**Sex: male**					
**Characteristics**	**Overall (n = 306) (I)**	**Patients with HIT suspicion (n = 44) (II)**	**Patients with excluded HIT (n = 32) (III)**	**Patients with confirmed HIT (n = 12) (IV)**	**p-value (III vs. IV)**
Total ICU length of stay [d], median [IQR]	9.1 [2.9, 16.0]	13.2 [7.7, 21.7]	9.4 [7.1, 15.9]	21.3 [16.2, 30.8]	0.005
Total hospital length of stay [d], median [IQR]	14.1 [5.0, 25.3]	27.1 [11.0, 48.3]	29.1 [9.5, 48.3]	27.0 [19.8, 37.2]	0.668
Hospital mortality, n (%)	173 (57)	18 (41)	13 (41)	5 (42)	>0.999

1-month mortality, n (%)		173 (57)	15 (34)	10 (31)	5 (42)	0.722
3-month mortality, n (%)		181 (59)	19 (43)	13 (41)	6 (50)	0.735
1-year mortality, n (%)		195 (64)	23 (52)	17 (53)	6 (50)	>0.999
Cerebral performance category of survivors on hospital discharge	CPC1, n (%)	16 (5)	4 (9)	4 (12)	0 (0)	0.562
	CPC2, n (%)	30 (10)	2 (5)	1 (3)	1 (8)	0.476
	CPC3, n (%)	61 (20)	11 (25)	8 (25)	3 (25)	>0.999
	CPC4, n (%)	26 (8)	9 (20)	6 (19)	3 (25)	0.687
**Sex: female**						
**Characteristics**		**Overall (n = 67) (I)**	**Patients with HIT suspicion (n = 9) (II)**	**Patients with excluded HIT (n = 8) (III)**	**Patients with confirmed HIT (n = 1) (IV)**	**p-value (III vs. IV)**
Total ICU length of stay [d], median [IQR]		8.7 [3.1, 16.8]	19.4 [18.6, 27.7]	20.5 [17.0, 28.8]	72.58	n.a.
Total hospital length of stay [d], median [IQR]		10.4 [4.0, 23.0]	25.9 [19.3, 53.4]	24.2 [19.3, 37.2]	72.58	n.a.
Hospital mortality, n (%)		40 (60)	4 (44)	4 (50)	0 (0)	n.a.
1-month mortality, n (%)		40 (60)	4 (44)	4 (50)	0 (0)	n.a.
3-month mortality, n (%)		41 (61)	4 (44)	4 (50)	0 (0)	n.a.
1-year mortality, n (%)		44 (66)	4 (44)	4 (50)	0 (0)	n.a.
Cerebral performance category of survivors on hospital discharge	CPC1, n (%)	3 (4)	1 (1)	0 (0)	1 (100)	n.a.
	CPC2, n (%)	7 (10)	2 (22)	2 (25)	0 (0)	n.a.
	CPC3, n (%)	14 (21)	2 (22)	2 (25)	0 (0)	n.a.
	CPC4, n (%)	3 (4)	0 (0)	0 (0)	0 (0)	n.a.

Supplementary Table S3: Adverse events during VA-ECMO therapy differentiated by gender.
BARC, bleeding academy research consortium; HIT, heparin-induced thrombocytopenia; n, number of patients; VA-ECMO, venoarterial extracorporeal membrane oxygenation.

**Supplementary Table S3: Adverse events during VA-ECMO therapy differentiated by gender**						
**Sex: male**						
**Characteristics**		**Overall (n = 306) (I)**	**Patients with HIT suspicion (n = 44) (II)**	**Patients with excluded HIT (n = 32) (III)**	**Patients with confirmed HIT (n = 12) (IV)**	**p-value (III vs. IV)**
**Adverse events during VA-ECMO therapy**						
Hemorrhage	BARC 3, n (%)	97 (32)	10 (23)	7 (22)	3 (25)	>0.999
	BARC 4, n (%)	1 (0)	0 (0)	0 (0)	0 (0)	
	BARC 5, n (%)	9 (3)	1 (2)	1 (3)	0 (0)	>0.999
Stroke, n (%)		13 (4)	1 (2)	0 (0)	1 (8)	0.273
Hemolysis, n (%)		37 (12)	8 (18)	5 (16)	3 (25)	0.663
Myocardial infarction, n (%)		9 (3)	0 (0)	0 (0)	0 (0)	

Arterial thrombosis, n (%)	18 (6)	5 (11)	3 (9)	2 (17)	0.603
Venous thrombosis, n (%)	10 (3)	3 (7)	1 (3)	2 (17)	0.176
Device related peripheral ischemic complications, n (%)	11 (4)	0 (0)	0 (0)	0 (0)	
Device malfunction, n (%)	3 (1)	0 (0)	0 (0)	0 (0)	
VA-ECMO oxygenator exchange, n (%)	5 (2)	1 (2)	1 (3)	0 (0)	>0.999
VA-ECMO circuit exchange, n (%)	10 (3)	5 (11)	4 (12)	1 (8)	>0.999
VA-ECMO oxygenator and circuit exchange, n (%)	2 (1)	0 (0)	0 (0)	0 (0)	
**Sex: female**					
**Characteristics**	**Overall (n = 67) (I)**	**Patients with HIT suspicion (n = 9) (II)**	**Patients with excluded HIT (n = 8) (III)**	**Patients with confirmed HIT (n = 1) (IV)**	**p-value (III vs. IV)**
**Adverse events during VA-ECMO therapy**					
Hemorrhage: BARC 3, n (%)	29 (43)	5 (56)	4 (50)	1 (100)	n.a.
Hemorrhage: BARC 4, n (%)	0 (0)	0 (0)	0 (0)	0 (0)	n.a.
Hemorrhage: BARC 5, n (%)	4 (6)	0 (0)	0 (0)	0 (0)	n.a.
Stroke, n (%)	3 (4)	1 (11)	1 (12)	0 (0)	n.a.
Hemolysis, n (%)	11 (16)	3 (33)	3 (38)	0 (0)	n.a.
Myocardial infarction, n (%)	0 (0)	0 (0)	0 (0)	0 (0)	n.a.
Arterial thrombosis, n (%)	3 (4)	1 (11)	1 (12)	0 (0)	n.a.
Venous thrombosis, n (%)	4 (6)	2 (22)	2 (25)	0 (0)	n.a.
Device related peripheral ischemic complications, n (%)	2 (3)	0 (0)	0 (0)	0 (0)	n.a.
Device malfunction, n (%)	2 (3)	0 (0)	0 (0)	0 (0)	n.a.
VA-ECMO oxygenator exchange, n (%)	2 (3)	1 (11)	1 (12)	0 (0)	n.a.
VA-ECMO circuit exchange, n (%)	3 (4)	1 (11)	1 (12)	0 (0)	n.a.
VA-ECMO oxygenator and circuit exchange, n (%)	2 (3)	1 (11)	1 (12)	0 (0)	n.a.
